# Prediction of Optimal Folding Routes of Proteins That Satisfy the Principle of Lowest Entropy Loss: Dynamic Contact Maps and Optimal Control

**DOI:** 10.1371/journal.pone.0013275

**Published:** 2010-10-12

**Authors:** Yaman Arkun, Burak Erman

**Affiliations:** Department of Chemical and Biological Engineering, Koc University, Istanbul, Turkey; National Institute for Medical Research, Medical Research Council, London, United Kingdom

## Abstract

An optimization model is introduced in which proteins try to evade high energy regions of the folding landscape, and prefer low entropy loss routes during folding. We make use of the framework of optimal control whose convenient solution provides practical and useful insight into the sequence of events during folding. We assume that the native state is available. As the protein folds, it makes different set of contacts at different folding steps. The dynamic contact map is constructed from these contacts. The topology of the dynamic contact map changes during the course of folding and this information is utilized in the dynamic optimization model. The solution is obtained using the optimal control theory. We show that the optimal solution can be cast into the form of a Gaussian Network that governs the optimal folding dynamics. Simulation results on three examples (CI2, Sso7d and Villin) show that folding starts by the formation of local clusters. Non-local clusters generally require the formation of several local clusters. Non-local clusters form cooperatively and not sequentially. We also observe that the optimal controller prefers “zipping” or small loop closure steps during folding. The folding routes predicted by the proposed method bear strong resemblance to the results in the literature.

## Introduction

Recent studies on protein folding lead to the suggestion that folding rates of two-state proteins are largely determined by a topological property of its three dimensional native structure [1], namely the contact order, CO, defined as the number of primary sequence bonds between contacting residues in space. Experiments show that folding rates of proteins decrease exponentially with average CO [1]. Structures with low CO such as helices, beta strands and tight turns fold fast. Structures with high CO, having non-local contacts between different substructures, like beta sheets and large loops fold slowly. In order to understand the mechanisms of folding, an additional independent parameter, the effective contact order, ECO, has been proposed for understanding the mechanism of folding [2], [3], [4], [5], [6]. In order to define ECO, we assume four residues *i*, *k*, *m*, and *j* along a primary sequence. We let *k* and *m* make a contact first. Then the CO is *k-m*. If *i* and *j* make a contact after the *km* contact is formed, then the ECO for the *ij* contact is the shortest path in the presence of the contact *km*. Thus ECO is a CO conditioned upon the prior contacts. Thus unlike CO, ECO is sensitive to the order in which contacts are formed, and hence an indicator of folding mechanisms or folding routes. ECO can be used to compute the entropy loss for loop closures or “zipping” during folding [5]. The hypothesis of zipping and assembly, ZA, has been successful in explaining the folding routes of several two state proteins [2], [3], [7]. According to the ZA hypothesis, the protein avoids searching the whole conformational space and essentially picks the low entropy loss routes (or low ECO routes) on a folding landscape [5]. The ZA hypothesis further postulates that the folding speed correlates with ECO. Thus, the knowledge of the contact map of the native state and the adoption of low entropy loss routes during folding are the two essential ingredients for understanding the sequence of events during the folding of a protein.

In this paper, we present a general optimization scheme to mimic the folding routes of proteins based on the prior knowledge of the native topology. The method assumes that folding takes place as a quasi-equilibrium process during which the protein has sufficient time to search for the minimum entropy loss routes. We show that as a consequence of the hypothesis of minimum entropy loss routes, while minimizing energy, the method correctly predicts the sequence of events during folding.

Ideally, the decrease of the Helmholtz potential of a system should result from a decrease of the energy and an increase of the entropy of the system. In the case of protein folding, however, both the energy and the entropy of a protein decrease during folding. Thus, there is an entropy penalty accompanying the folding process, and it is expected that in the interest of efficiency, nature diminish this penalty. The formation of a native or a non-native contact imposes constraints on the conformation of the protein. In this respect, folding may be approximated by a succession of constrained equilibrium states. At each addition of a constraint to the system, the entropy decreases. Thus, folding has to progress along entropy loss routes. Several such routes are possible on the folding landscape, starting from a given initial state and ending in the folded state. Our optimization method computes the minimum-energy routes that try to avoid high entropy loss that leads to inefficient folding pathways.

## Methods

### The general thermodynamics basis of the optimization problem

**Figure 1:**
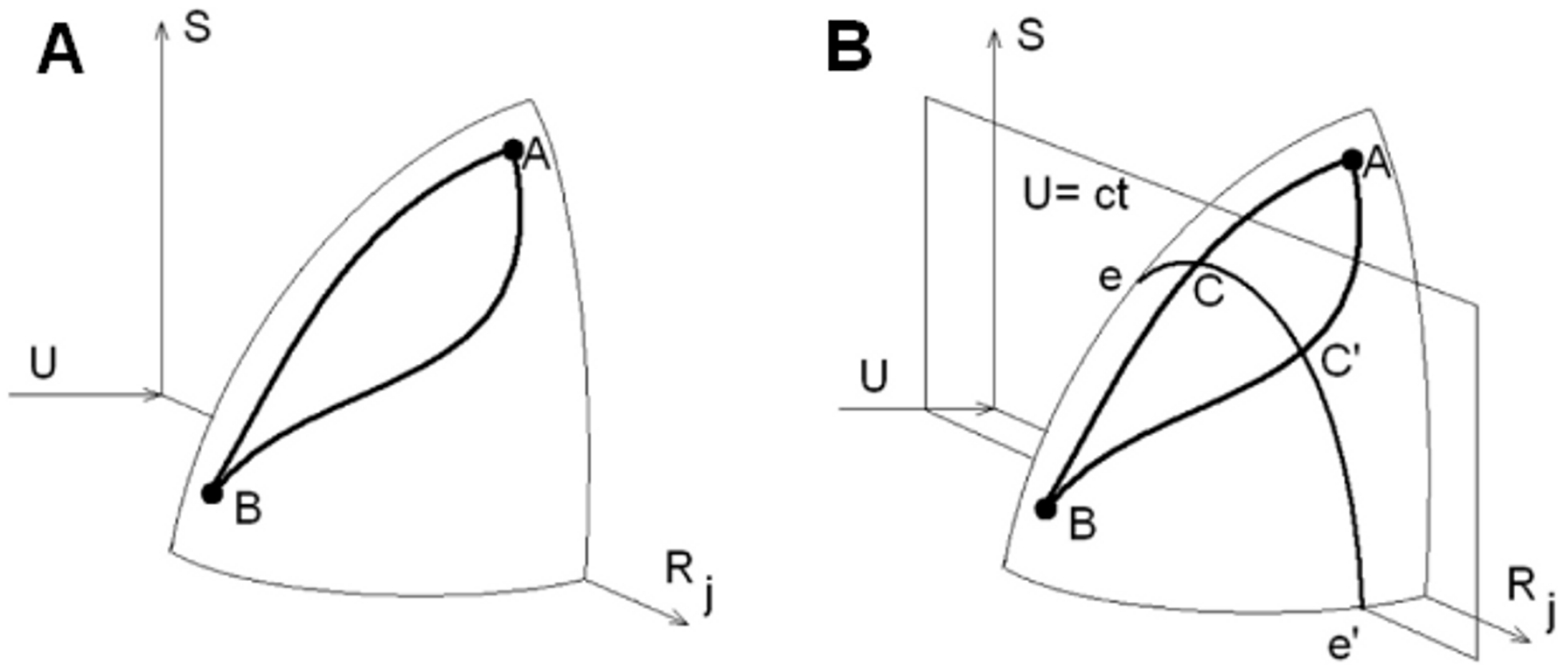
The thermodynamic surface and two routes of folding. Point A represents an initial state and point B is the folded native state. Two paths between A and B are shown (panel A). Two paths ACB and AC′B on the thermodynamic folding surface. U = ct denotes the constant energy surface (panel B). Paths AC and AC′ correspond to the same energy loss from the starting point A, but point C corresponds to a smaller entropy loss than C′.

In the coarse grained model of the protein, the equilibrium states of a protein of *n* residues is represented by the thermodynamic fundamental equation which expresses the entropy *S* as a function of energy *U* and positions 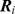 of the ith alpha carbon, 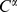, as [8]

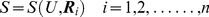
(1)Equation 1 defines a hypersurface [8] which is schematically shown in Figure 1. In this figure, 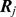 is just a symbolic representation of the jth conformation. Point A represents an initial state and point B is the folded native state. Two paths between A and B are indicated on the surface. Both the energy and the entropy decrease along these paths, as the protein moves from A to B. These are equilibrium paths, on which the protein goes through a succession of equilibrium states. In this paper, we assume that folding takes place quasistatically, and the surface indicated in Figure 1 is a good representative of the folding process.

The energy of the protein decreases as its residues make favorable contacts. We assume that the protein goes through a succession of such favorable contacts until the native state is obtained. In Figure 1, the constant energy surface U = ct intersects the hypersurface along the curve eCC′e′. The paths AC and AC′ correspond to the same energy loss from the starting point A, but point C corresponds to a smaller entropy loss than C′. In fact the path ACB is chosen such that it corresponds to maximum entropy at every constant energy surface that intersects the hypersurface. Stated in another way, the path ACB is the lowest entropy loss path for folding. All other routes correspond to higher entropy losses during folding. At U = ct, points C and C′ correspond to different sets of favorable contacts, leading to the differences in the entropy. Each set corresponds to a constrained state of the protein at that energy. The set with least unfavorable constraints is the smallest entropy loss route. With the proposed method, we aim at generating small entropy loss routes.

The change 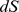 in entropy is obtained as the differential of Eq. 1
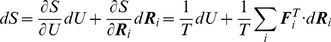
(2)Here, 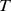 is the temperature and the force vector 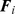 is obtained from the thermodynamic expression 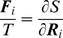. The forces defined in this way are general, and may further be specialized to represent the various effects on the residues, such as external forces, forces coming from excluded volume effects, etc.

The Euler form of Eq. 2 is
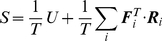
(3)We use a coarse-grained model to describe the protein chain where each amino acid residue is represented with spherical beads centered at the 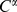 atoms. The number of such beads is equal to *n*. The position vector of the i-th 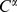 atom is represented by 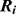.

The total position vector 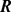, whose ith entry is the position vector 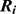, obeys the equation of motion:
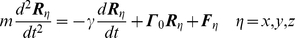
(4)where, the subscript 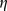 denotes the x, y, or z coordinates, *m* is the mass of the ith residue and 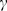 is the local friction force with dimensions of force-time/length and 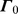 is the connectivity matrix of the initial structure, defined similar to that of the Gaussian Network Model [9], [10].

It is to be noted that Eq. 4 is a deterministic equation in the sense that the forces 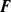 are not random but determined by the optimization scheme. Ignoring the mass term and expressing the variables in deviation from their native state values leads to
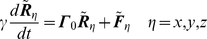
(5)By construction 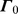 has *n-1* negative and one zero eigenvalue. In the following, with slight abuse of notation, we omit using the subscript 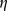 that refers to the *x*, *y* or *z* coordinates.

The Euler form of entropy Eq. 3 can now be expressed for Eq. 5 in terms of deviation variables and both sides of Eq. 3 can be integrated from 0 to the final time 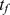 to give:
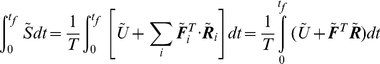
(6)where 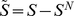, 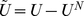, 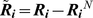, and 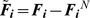, and the superscript *N* denotes the native state.

In general, under the integral given by Eq. 6, the energy 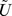 and the forces 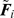 are complicated functions of residue positions. In the interest of simplifying the model, we make the harmonic assumption for the energy
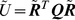
(7)which represents the excess potential about the native state. ***Q*** is a positive definite matrix.

Its exact form will be described in the sequel.

The vector 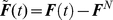 represents the forces acting on 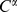 atoms with the following properties:

- In the native state, 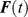 is a steady-state force field 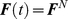 which keeps 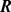 at 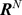. Thus, Eq. 4 gives, 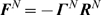, where now 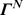 is the connectivity matrix of the native structure. Without this constant force field, the chain would collapse to zero volume. Thus at the native state excluded volume constraints are satisfied by imposing 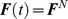.
- When the position vector deviates from its native state i.e., 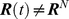, 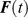 must also deviate from its native state to bring the position vector back to its native state. Thus, the total force field is 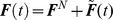 in which 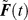 is computed optimally as described next.

The optimization problem may now be stated as follows: The protein tends to escape high energy regions of the energy landscape during its excursion to the native state. Thus, we have to minimize the energy of the protein:
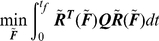
(8)where the dependence of 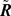 on the force field through the equation of motion [6] is explicitly shown.

If there were no constraints in this problem, both 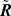 and energy would decay to zero infinitely fast under an unrealistic, unbounded force field 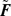. Such folding routes would violate the principle of minimum entropy loss. Therefore, we enforce the following entropy constraint, which must be obeyed by the optimal solution of the above minimization:
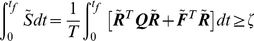
(9)where, constant 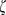 is the desired lower bound for the cumulative entropy during folding. The purpose of this constraint is to prevent fast decay of excess entropy 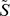 that is associated with high entropy loss routes. The solution of this constrained problem gives folding trajectories on the energy landscape such that the curve AC′B in Figure 1b approaches the curve ACB.

We prefer to solve the above constrained minimization problem by converting it to a well-known optimal control problem whose closed-form solution is straightforward and well-characterized. This is done at the expense of some suboptimality but the form of the optimal solution facilitates the understanding of the folding process and allows a closer comparison with the literature results. In doing so, the thermodynamic basis of the original problem expressed by Eqs. 8 and 9 is not lost as discussed below.

### Optimal Control Formulation: Linear Quadratic Regulator

Our dynamical system is modeled by:
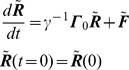
(10)The Linear Quadratic Regulator (LQR) computes an optimal feedback solution for the input 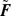 that brings the initial state to the zero-steady state satisfying some prescribed desired dynamic performance. Since the variables are in deviation, the zero-steady state corresponds to the native state in our case. The following minimization is solved subject to Eq. 10:
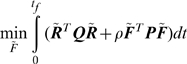
(11)The weighting matrices ***Q*** and ***P*** are non-negative definite and positive definite symmetric matrices. Both matrices are pre-specified and depend on the physics of the problem. The parameter 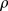 is used as a tuning parameter to reflect the relative importance of the two terms under the integral. As the terminal time 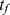 approaches infinity, the optimal solution of the above problem is given by a negative constant feedback control law [11]:
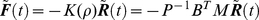
(12)where 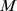 is positive definite and it is easily computed from the algebraic Riccatti equation [11].

We note that the optimal feedback gain *K* is positive definite (i.e. 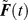 is attractive); it is independent of the initial condition 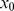 and depends strongly on the tuning parameter 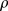; thus, denoted by 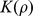.

The objective that is minimized by LQR is thermodynamically consistent with that of the original optimization represented by Eqs. 8 and 9. The first term under the integral in Eq. 11 is the energy. The second term that is minimized expresses the cost incurred if high entropic routes are followed. The parameter 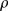 acts like a Lagrange multiplier to penalize costly entropy loss routes and thus helps to enforce the inequality 9. This effect can be seen more clearly as follows.

Assume that the optimal solution of the original thermodynamics based optimization can be parameterized by the optimal LQR solution i.e. 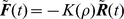. Substituting this into Eq. 11:
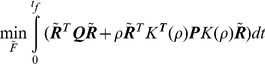
(13)Similarly, substituting it into the entropy constraint gives:
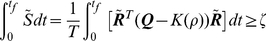
(14)An important property of LQR solution is that by increasing 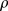, 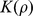 can be made sufficiently small [11].

For a higher value of the tuning parameter 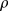, minimization given by Eq. 13 places more emphasis on the second term under the integral and reduces the magnitude of 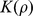 since it wants to minimize the second term. This is the same as penalizing the high entropy loss routes, because lower 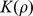 values are favored by the entropy constraint. Specifically, for a given 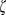, the entropic constraint can be satisfied by a sufficiently small 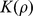 which makes the left hand side of inequality 14 sufficiently large.

**Figure 2:**
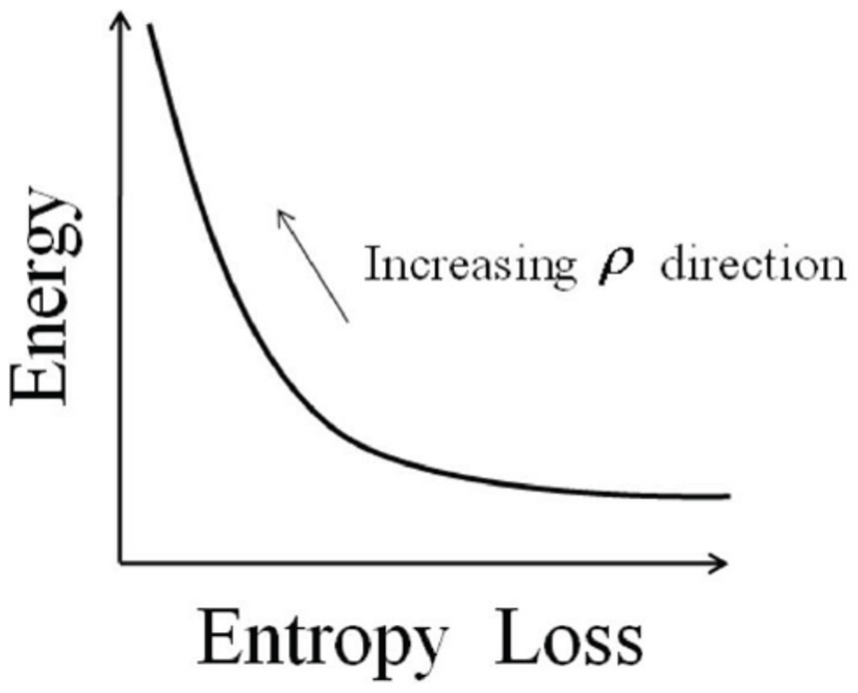
Energy versus entropic penalty terms of the integral (13) as a function of the tuning parameter 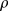. The curve indicates the possible values of energy and entropy loss depending on the choice of 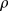.

In summary, the proposed method uses Eq. 13 to evade the high energy regions of the landscape while choosing entropically favored folding pathways by penalizing high entropy loss routes. It should be noted that the optimal solution is a trade-off between how much energy is minimized (first term) and how much high entropy loss can be avoided (second term). One cannot be improved without worsening the other. This is illustrated in Figure 2. By choosing 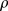 appropriately a compromise can be established in which energy is minimized with a constrained entropy loss.

### Implementation of the linear quadratic optimal control algorithm

We solve the LQR minimization denoted by Eq. 11 subject to the dynamic model Eq. 10. The weighting matrices ***P*** and ***Q*** have to be specified to implement this optimization. Without any loss of generality, we take ***P*** as the identity matrix *I* which means that each component of the force vector 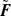 contributes equally to the objective function. ***Q*** emerges from the Contact Map (without the covalent bonds) as shown below.

Let 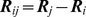 denote the vector from residue *i* to residue *j*. Also let its deviation from the native state be denoted by 
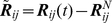
. Recalling 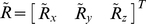, the following relationship holds:
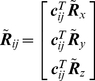
(15)where 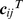 is a 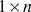 row vector whose i-th element is −1 and the j-th element is 1. The row vector 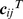 operates on 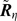 by subtracting its i-th entry 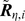 from its j-th entry 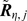. The square of deviations from the native state for the pair i-j follows from Eq. 15:
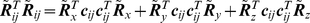
(16)Summing up the squares of the deviations over all the pairs, one gets:
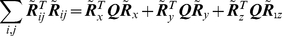
(17)where
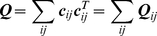
(18)By this construction
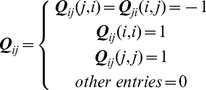
(19)The contact map of a protein is an *n*x*n* matrix defined as follows:
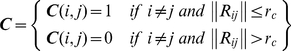
(20)The parameter 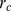 is the cut-off distance (e.g. 7 Å) for a contact to be established between two residues.

The Laplacian matrix [12], [13], [14] is an *n*x*n* matrix constructed from the contact map ***C*** as follows:
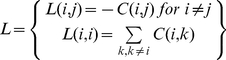
(21)With the above definitions and properties, it immediately follows that matrix ***Q*** is equal to the Laplacian matrix excluding the covalent bonds i.e.
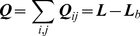
(22)where 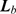 is the Laplacian matrix consisting of the covalent bonds only.

In our original model Eq. 5 the connectivity matrix 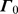 has all negative eigenvalues but one zero eigenvalue. This zero eigenvalue needs to be stabilized by the optimal controller so that the protein asymptotically can reach its native state. To do so ***Q*** must be positive definite; otherwise, no stabilizing optimal feedback gain matrix *K* exists. However, by definition, the Laplacian matrix has all positive eigenvalues but one zero eigenvalue. Therefore setting ***Q*** equal to 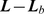 violates the positive definiteness requirement. For this reason we modify ***Q***:
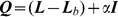
(23)where 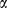 is a small positive number. The free parameters of the minimization given by Eq. 11 are 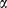 and 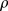 which are used as tuning parameters and their effects are well-understood (see below).

### Properties of the Optimal Solution

The structure of 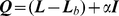 imposes a similar topology on the optimal gain matrix *K*. Therefore *K* can be similarly decomposed as:
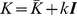
(24)where 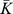 is the “harmonic spring constant matrix” since its row sums are all equal to zero. In the second term *k* is a scalar.

Under the action of this optimal control 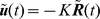, the equation of motion Eq. 5. becomes:
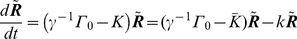
(25)It is seen from Eq. 25 that the folding dynamics is governed by a Gaussian network with the connectivity matrix 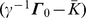 where 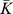 represents the set of springs added to the original connectivity matrix 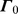. Thus the linear quadratic regulator synthesizes both the optimal network topology (i.e. through the topology of 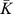) and the strength of the network connections (represented by the values of matrix elements of 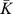).

In addition to the pairwise connections, each residue is connected to its native state so that the translational mode or the zero eigenvalue is stabilized. The second term with *k* in Eq. 25 contains these *n* connections that anchor the network. Optimal controller assigns the same strength or spring constant *k* to each of these connections.

### Role of the Parameters

The value of the parameter 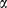 determines the magnitude of the second term *k* ***I*** in Eq. 24. In our simulations we assign a small value to 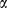 indicating that the residues are connected to their native states with weak springs and folding dynamics is dominated by the pairwise interactions. As explained earlier, 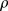 is the most critical tuning parameter and is used to establish a compromise between energy minimization and entropy loss.

### Implementation of the Method Using the Dynamic Contact Map

We assume that the native state is known and the model is given by Eq. 5 where all the parameters are specified. The choice of the weighting matrix ***Q*** is critical in defining the optimal folding path. In our implementation, ***Q*** is updated depending on the contacts made during folding. At the beginning of the simulations, ***Q*** is initialized with 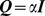. Using this ***Q***, optimization computes the optimal *K*; and equation of motion Eq. 25 is next simulated with this *K* value. Next at some future sampling time 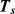 in the early stages of folding, we measure the contacts made and construct the contact map ***C***. From this contact map, the Laplacian matrix ***L*** is computed using Eq. 21 and ***Q*** is updated according to 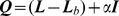. Optimization-simulation cycle is repeated after each update of ***Q*** at 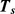 time intervals until the protein folds to its native state. When the protein reaches the native state, the sequence of dynamic contact maps i.e. 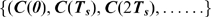 converges to the native contact map. This is a learning optimal control algorithm whereby the Gaussian network is slowly learned as contacts are made at each folding step and the entries of the contact map are filled dynamically along the optimal folding trajectory. The way the contact map is built describes the sequence of the time-ordered folding events which we next analyze.

## Results

**Figure 3:**
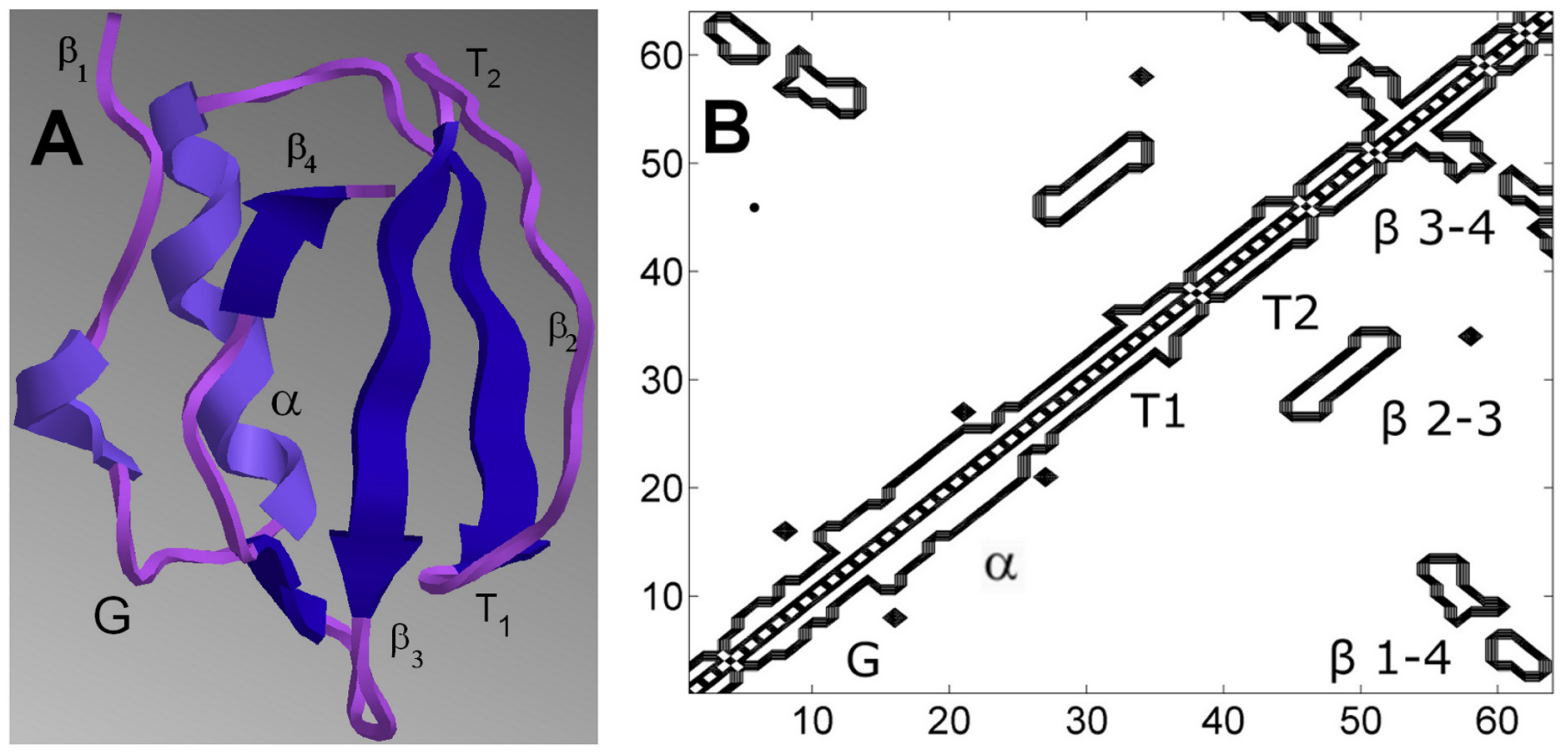
CI2 (panel A) and its native contact map contours (panel B). The 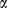-helix, turns T1 and T2, the 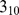-helix G, and the 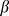 strands 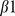, 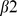, 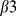 and 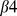 are indicated on the figure.

The first example is chymotrypsin inhibitor 1, CI2, (PDB code 1YPA) whose folding has been characterized extensively (see e.g. [15], [16], [17], [18], [19], [20], [21]). CI2 with its four-stranded 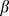-sheet and 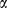-helix structure is shown in Figure 3 along with its native contact map.

**Figure 4:**
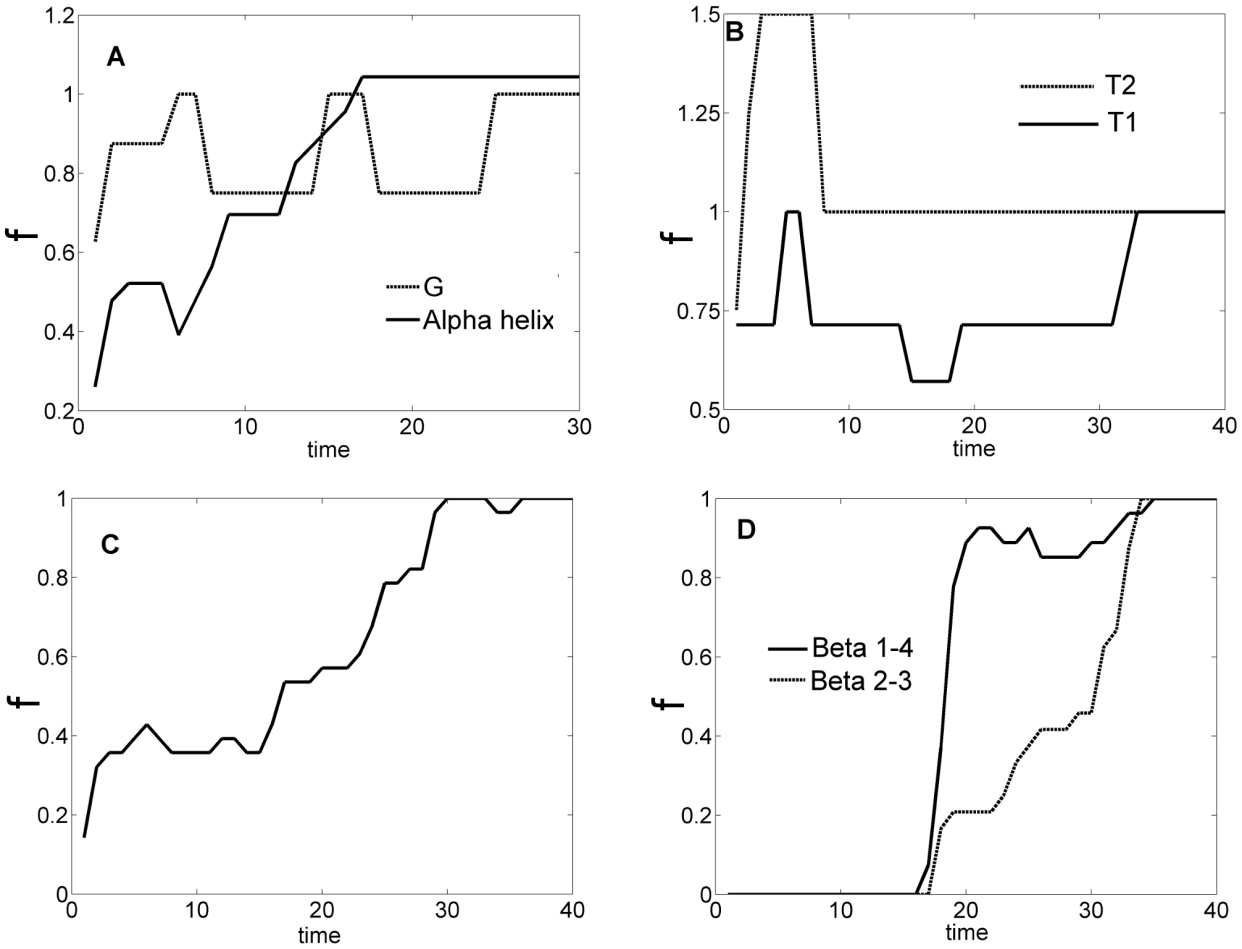
The fractional completion of CI2 contacts versus folding time. A. Helices, B. Turns, C. 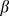
3–4, D. 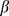
1–4 and 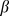
2–3. The ordinate, 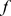, expresses the ratio of the contacts formed to those of the native structure. T2 and the 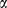-helix form first. The local cluster 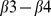 and T1 form next. Formation of the non-local clusters requires these local clusters to form first, which in turn reduces ECO. Local clusters are followed by the non-local clusters 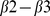 and 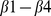.

As shown on the contact map, CI2 has five local clusters: 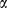-helix, turns T1 and T2, a 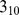-helix G, 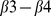 and two non-local clusters: 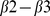 and 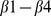. The order of the formation of contacts is illustrated in Figure 4. The ordinates, 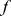, denote the ratio of the contacts formed to those of the native structure. T2 and the 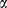-helix form first at t = 10 and t = 20, respectively. This is followed by the formation of the local cluster 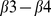 at t = 30 and T1 at t = 35, respectively. Formation of the G helix is initiated early but its complete formation (*f* = 1) takes about the same time as 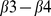. Once these local clusters form, ECO decreases and formation of the non-local clusters is facilitated. This confirms the observation made by [5] in that non-local clusters generally require the formation of several local clusters. 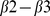 contacts are initiated after the two turns sufficiently form as concluded in [5] as well. Non-local clusters 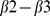 and 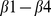 form cooperatively after an initial delay but not sequentially as they start and complete their formation at the same times. The folding route provided by our optimal controller prefers contacts that are easier to make with smaller entropy barrier as in the case of following routes with smallest ECO in [5]. We also observe the same kind of “zipping” or small loop closure steps during folding. The values of 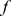 for T1 exceeds unity significantly in the initial stages of folding (see the second panel of Figure 4), indicating the presence of non-native contacts.

**Figure 5:**
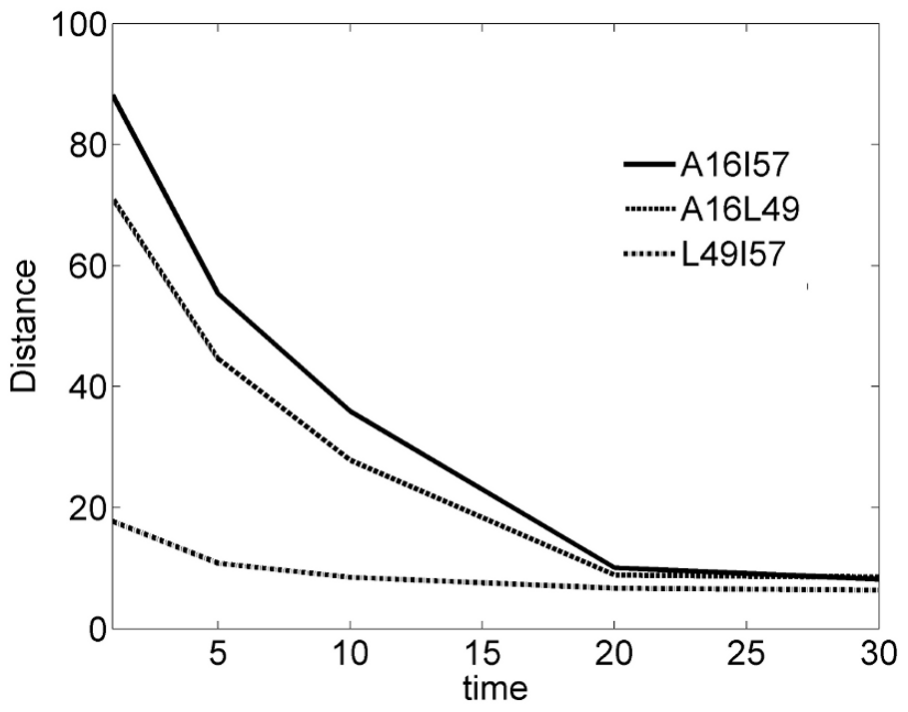
The evolution of the nucleation site. The solid, dotted and the light dotted curves represent the distances between ALA16-ILE57, ALA16-LEU49 and LEU49-ILE57.

In the literature, a nucleation site of CI2 that includes regions around ALA16, LEU49 and ILE57 has been noted [17]. The evolution of this core in terms of distances among the residues as function of folding time is shown in Figure 5. The trends and numbers are similar to those given in [17]. The curves approach each other and pack closely beyond time t = 20.

**Figure 6:**
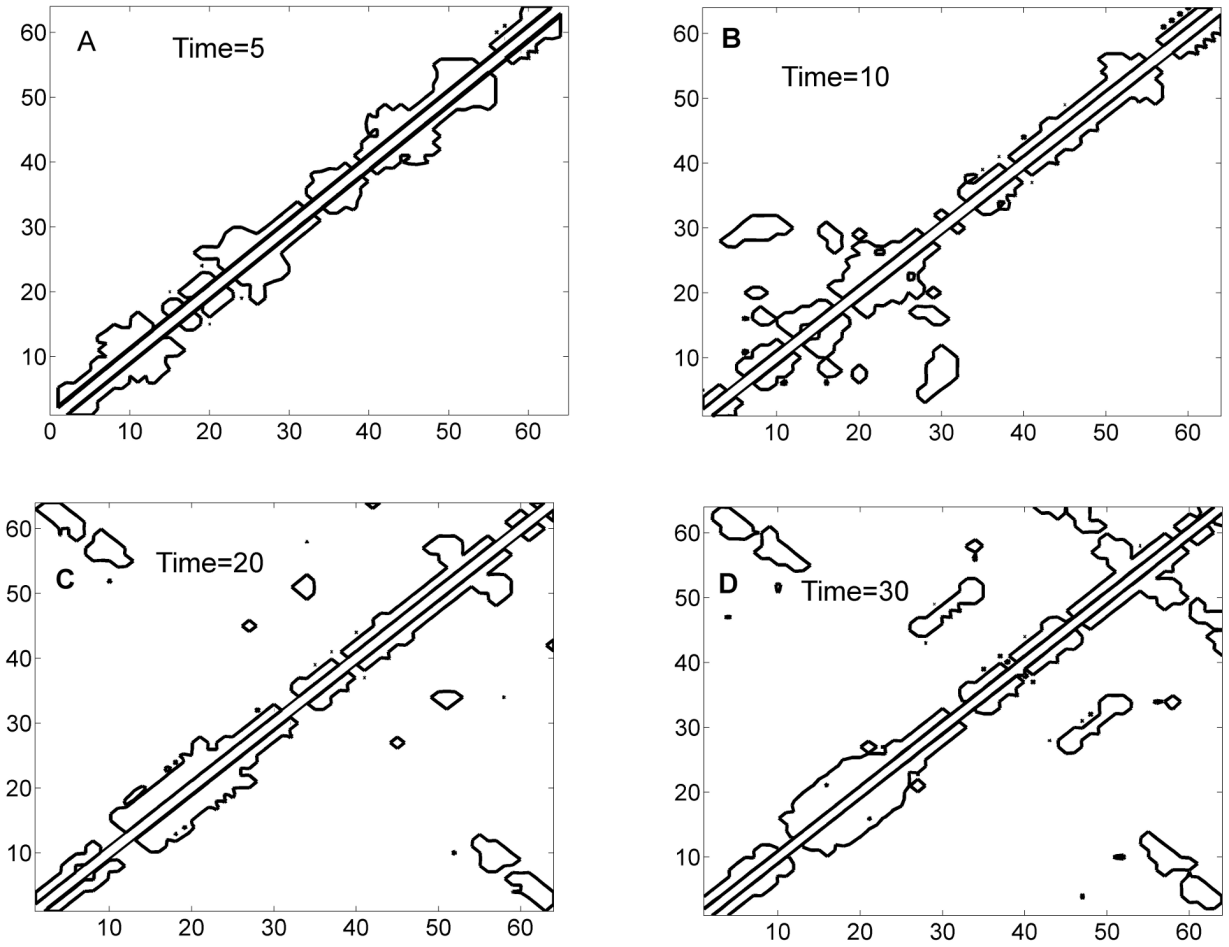
Dynamic evolution of the contours of Gaussian Network matrix 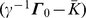. The Gaussian Network matrix 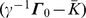 changes as optimal 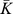 varies during folding. Four snapshots are given at four different folding times to show the dynamic evolution of the network topology. The network topology and the dynamic contact map evolve in a similar fashion.

The optimal solution focuses first on nearby local contacts. Thus, among the energetically favored folding pathways it prefers to synthesize routes with less entropy loss. This is also revealed by the fact that in early part of the folding process the optimization avoids to compute the connections in the spring constant matrix *K* that correspond to non-local interactions. These entropically more expensive distant connections are established after the local interactions are made. This is shown in the dynamic evolution of the topology of the optimal Gaussian network 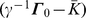 as the optimal controller 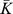 is synthesized and added to the backbone 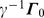 (see Figure 6).The corresponding contact maps evolve in a similar fashion which are not repeated here.

**Figure 7:**
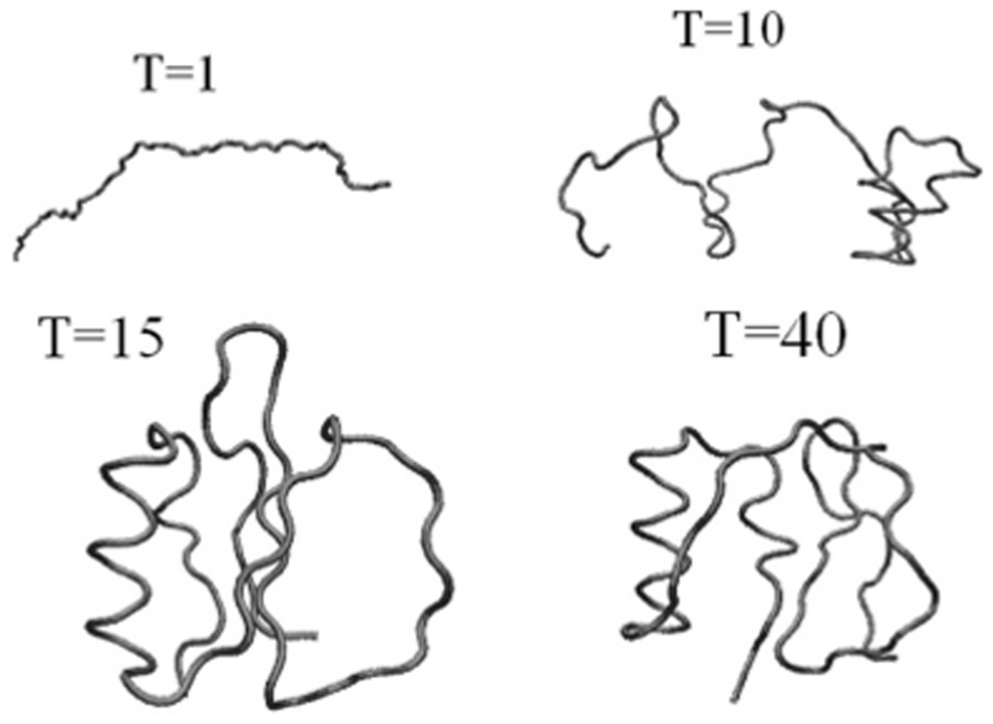
CI2 configurations at four different folding times. Earlier formation of local clusters (helices, turns, and 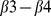) is followed by the nonlocal clusters 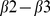 and 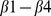.

Snapshots of configurations at different sampled folding times are shown in Figure 7.

**Figure 8:**
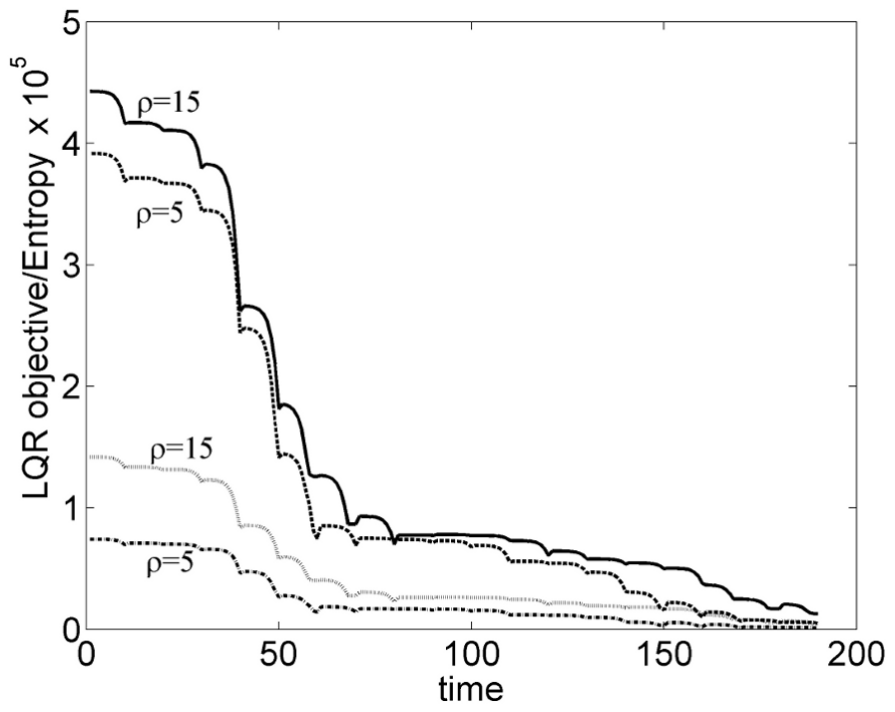
Comparison of LQR objective (upper two curves) and the entropy (lower two curves) for two values of 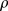. Both energy and entropy decrease along the folding pathways. Smaller value of the optimization tuning parameter 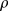 gives a faster decay of energy but at the expense of higher entropy loss.

**Figure 9:**
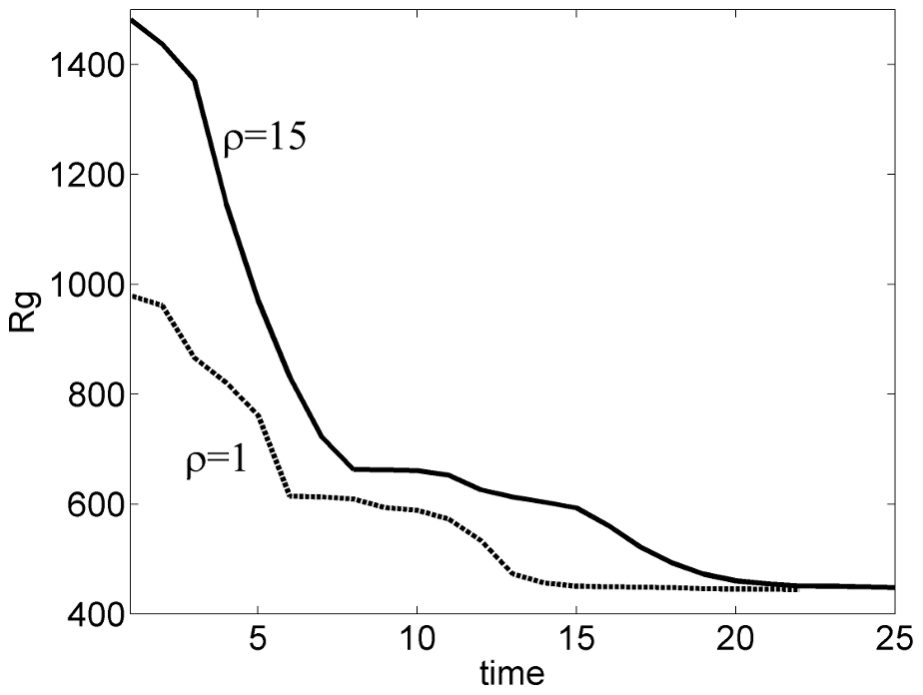
Effect of 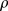 on the radius of gyration. Smaller value of the optimization tuning parameter 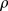 gives a faster decay of the radius of gyration to its final native state value.

The LQR objective that has been minimized, Eq. 13, and entropy constraint, Eq. 14, are compared in Figure 8 for two different values of 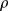. The folding pathways were found to be similar for both cases. The results indicate that both energy and entropy decrease along the folding pathways; and by tuning 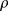, the decay of entropy can be maintained above a desired threshold. Radius of gyration, 
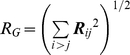
 plots in Figure 9 also indicate a similar effect of 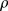. The decay rate of the radius of gyration and thus the folding rate can be tuned to reflect reality without altering the sequence of events.

**Figure 10:**
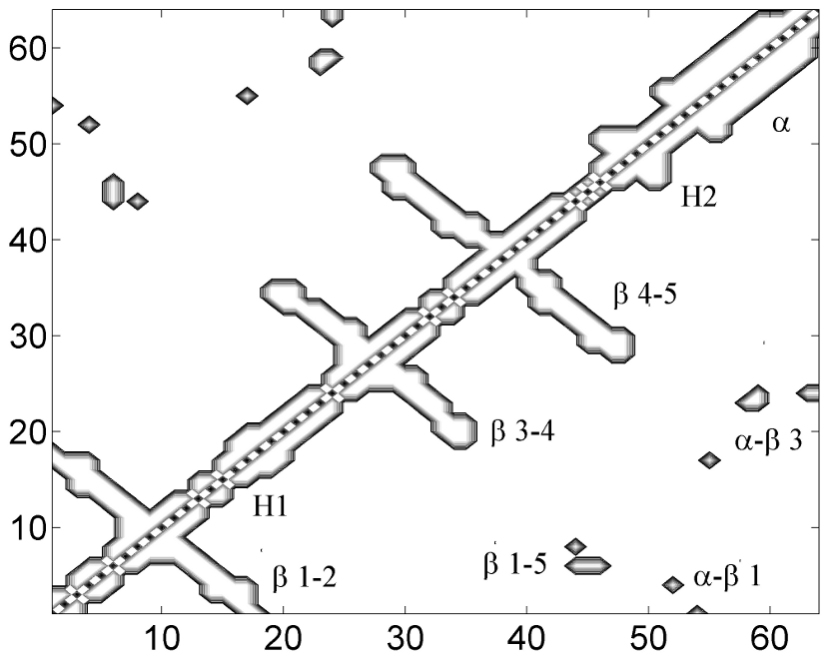
Native contact map contours of Sso7d. The local clusters are marked as a 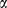 helix, 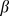
1–2, 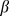
3–4, 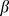
4–5, H1 and H2. Non-local clusters are marked as 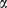-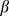
3, 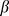
1–5 and 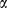-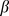
1.

For the second example we have used a DNA binding protein Sso7d (pdb code 1BNZ) which has 64 residues [5]. Its native contact map with helices and beta strands is shown in Figure 10.

**Figure 11:**
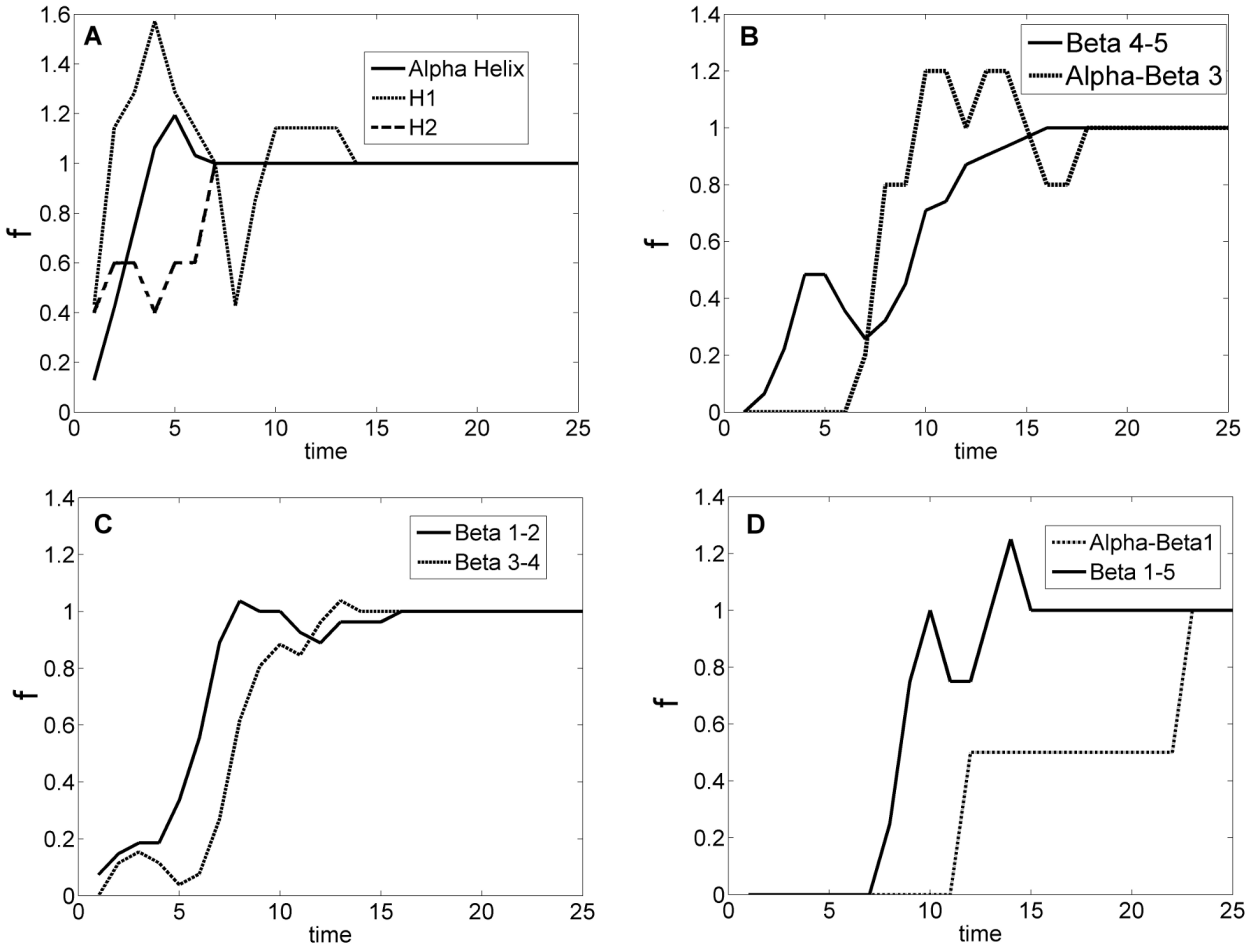
Fraction of native contacts made as a function of folding time. The ordinate, 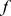, expresses the ratio of the contacts formed to those of the native structure. Contacts first start to form in the local clusters which are 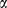 helix, 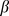
1–2, 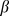
3–4, 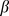
4–5, H1 and H2. These contacts are followed by the formation of non-local clusters 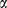-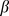
3, 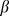
1–5 and 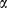-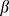
1. Complete formation of 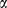-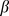
1 takes longest time and is completed sequentially after the contacts in 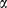-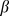
3 and 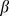
1–5 are established.

Formation of native contacts during folding is given in Figure 11. Contacts first start to form in the local clusters which are alpha helix, Beta 1–2, Beta 3–4, Beta 4–5, H1 and H2. These contacts are followed by the formation of non-local clusters alpha-Beta 3, Beta 1–5 and alpha-Beta 1. Complete formation of alpha-Beta 1 takes longest time and is completed sequentially after the contacts in alpha-Beta 3 and Beta 1–5 are established. This folding route or the sequence of folding events is consistent with the results presented in Weikl and Dill [5].

**Figure 12:**
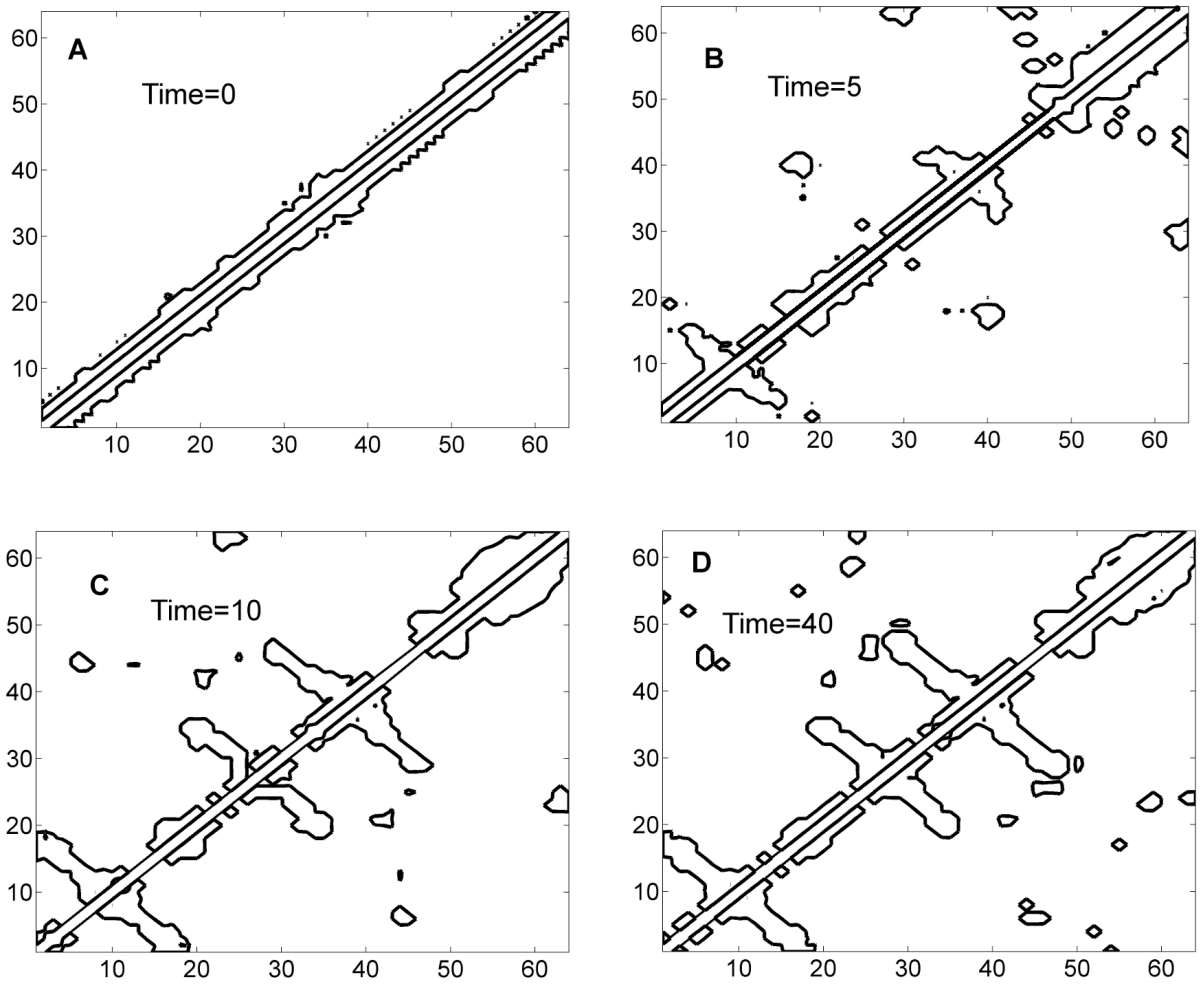
Dynamic evolution of the contours of the Gaussian Network matrix 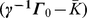. The optimal Gaussian Network matrix 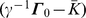 gets updated as new values of the “harmonic spring constant matrix” 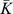 are computed by the optimal controller at different folding times indicated on the figure.

As optimal controller makes contacts (or loop closures), the Gaussian Network gets updated as shown in Figure 12. Contact map is filled in a similar fashion starting with local contacts

The last example is the Villin headpiece which is a 36-residue fast folding protein. Following the work of Duan and Kollman [22], the folding dynamics of Villin has been studied extensively (e.g. see 21,22).

**Figure 13:**
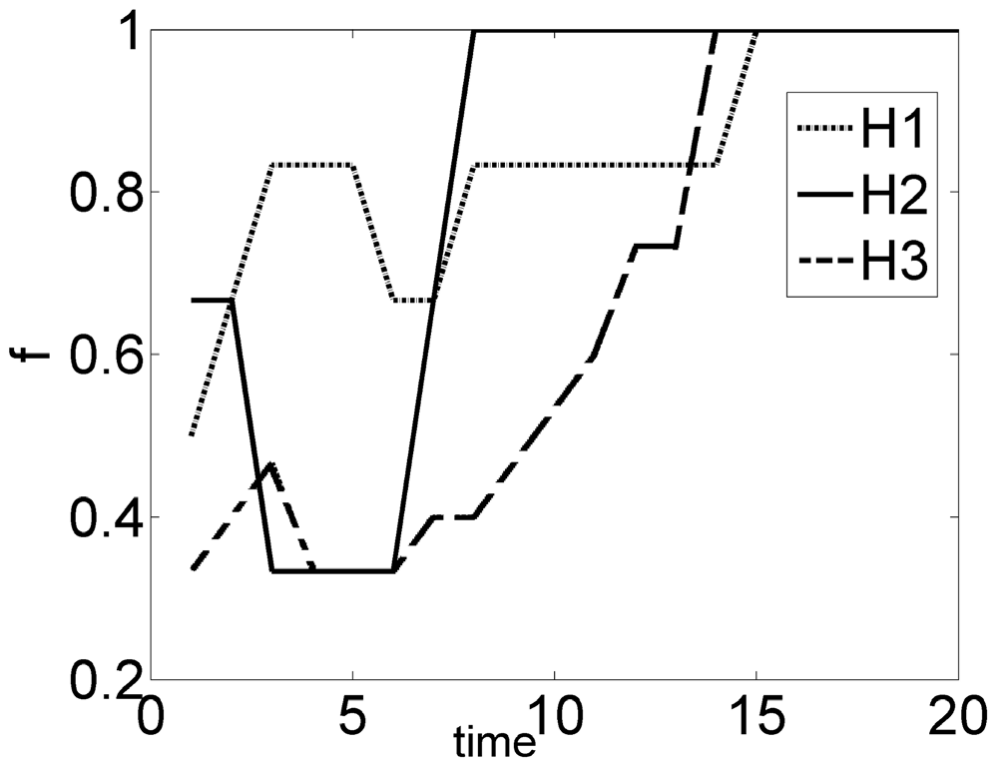
Fractional contacts of helices. Partial formation of H1 occurs first. The smallest helix H2 completes its formation at around a critical time = 7. The biggest helix H3 starts forming along with H2 and H1 but at slower pace. H3 formation accelerates due to several nonlocal tertiary contacts.

As seen from Figure 13, first a partial formation of H1 occurs (with fractional contact = 0.83). The smallest H2 is the first helix to complete its formation at around a critical time = 7. The biggest helix H3 starts forming along with H2 and H1but at slower pace. After about time = 7, H3 formation accelerates due to several nonlocal tertiary contacts. These long-range native contacts are initiated later than the local contacts as shown in Table 1.

**Table 1:** Contacts and their initial formation times.

Contact initiation time	Contacts
7–9	10–34,10–33,11–34,11–33
10	7–34
18	1–34

Helix 3, Helix 1 and the tertiary structure are established concurrently. In our simulations we have also observed non-native contacts between the loop residues (10, 11) and H3 residues (26,27) during times 7–9 which increases the compactness and the concurrent formation of H3. These observations are similar to the results in the literature that helical secondary structure and tertiary contacts are concurrently formed after a hydrodynamic collapse [23].

**Figure 14:**
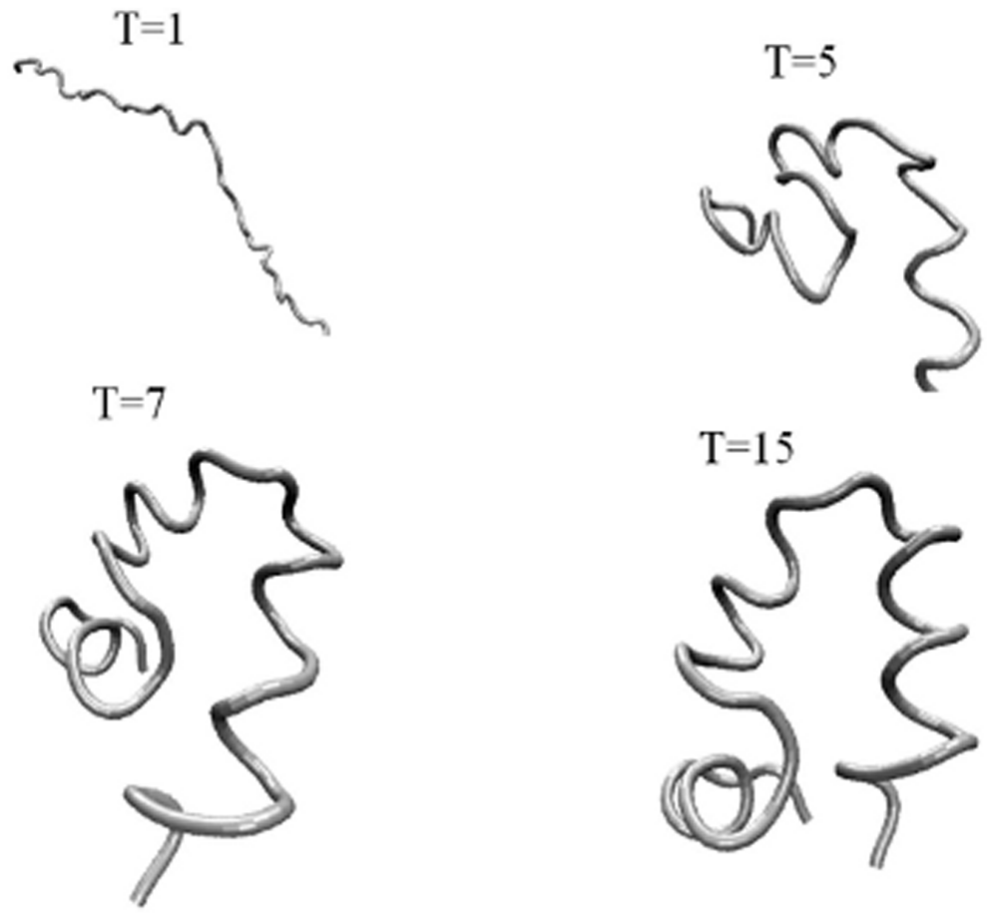
Snapshots of Villin conformations at four sampled times. Snapshots of configurations are taken at four different times to demonstrate the folding process. Local nearby contacts are formed first followed by long contacts in order to preserve low entropy loss during folding.

Figure 14 gives the snapshots that demonstrate the folding process. Like in the previous two examples the optimal Gaussian network establishes local nearby interactions first followed by long contacts in order to preserve low entropy loss during folding.

## Discussion

Many different types of folding mechanisms (e.g. zipping and assembly, hierarchical ordering, nucleation-condensation, diffusion-collision) exist in the literature [24]. Two general principles are used extensively to describe the folding pathways on the energy landscape [5], [25]: During folding, proteins (i) try to evade high energy regions of the folding landscape, and (ii) prefer low entropy loss routes. Using these two principles, and the contact map information of the native state, we formulated the prediction of folding trajectories as a dynamic optimization problem that has a thermodynamic basis. The problem is parameterized and solved within the framework of optimal control whose easily accessible solution provides a practical insight into folding dynamics. There is ample evidence in the literature that the protein's folding route or sequence of events evolves depending on the prior contacts made. To this end, we have introduced the notion of dynamic contact map. During the course of folding, the optimal solution is updated as the dynamic contact map changes from its initial given state to the final native state. As an important side product, optimal solution synthesizes the Gaussian Network topology that governs the optimal folding dynamics. Solution of the dynamic model under the action of this network gives the sequence of events during folding which we further analyze. To the best of our knowledge such a dynamic optimization formalism, founded on both thermodynamics and optimal control principles that recognize the physics of folding through dynamic contact maps, is first of its kind. Finally, computations are very fast since we take advantage of the machinery of a well-known optimal control algorithm i.e. linear quadratic regulator.

Our simulation results on three proteins CI2, Sso7d and Villin elucidate that folding starts by the formation of local clusters. Non-local clusters generally require the formation of several local clusters. Non-local clusters form cooperatively and not sequentially. We also find that the optimal controller provides “zipping” or small loop closure steps during folding. This important observation supports the previous work of Dill and collaborators [5] on the folding lansdscape. Entropically unfavorable distant connections are established after the local interactions are made.

The proposed optimization includes an entropy constraint, which penalizes the contacts that include high entropy losses. Accordingly, the decay rate of entropy, radius of gyration and folding rate can be affected. In this context, we are able to control the excluded volume constraints on an average sense. However, there is no guarantee that excluded volume constraints will not be violated at the residue level. In fact, in the first two examples we observed temporary isolated violations of excluded volume among some of the residues. However, the fact that our predicted folding routes are similar to the literature results indicates that the method is robust to these potential violations. Therefore, including excluded volume constraints among all residues explicitly into the optimization may not warrant the additional complexity. For the smaller protein, the Villin headpiece, we were able to perform such computationally demanding constrained optimizations [26]. We found that the folding routes were similar to those predicted in this paper which further supports the reliability of the proposed method. Nevertheless, it remains to be seen in general how the characteristics of the folding routes would change, if at all, when excluded volume constraints are individually accounted for in the optimal control formulation.
